# Exploration of two methods for quantitative Mitomycin C measurement in tumor tissue *in vitro* and *in vivo*

**DOI:** 10.1186/1480-9222-15-12

**Published:** 2013-11-09

**Authors:** Lee MacKenzie Fischer, Juan Luis Vásquez, Julie Gehl, Gregers G Hermann, Niels B Larsen

**Affiliations:** 1 Technical University of Denmark, DTU Nanotech - Department of Micro- and Nanotechnology, Ørsteds Plads 345Ø, 2800 Kongens Lyngby, Denmark; 2 Department of Oncology, Center for Drug and Gene Electrotransfer, Copenhagen University Hospital Herlev, Herlev Ringvej 75, 2730 Herlev, Denmark; 3 Department of Urology, Copenhagen University Hospital Frederiksberg, Nordre Fasanvej 57, 2000 Frederiksberg, Denmark

## Abstract

Two methods of quantifying Mitomycin C in tumor tissue are explored. A method of ultraviolet-visible absorption microscopy is developed and applied to measure the concentration of Mitomycin C in preserved mouse tumor tissue, as well as in gelatin samples. Concentrations as low as 60 *μ*M can be resolved using this technique in samples that do not strongly scatter light. A novel method for monitoring the Mitomycin C concentrations inside a tumor is developed, based on microdialysis and ultraviolet-visible spectroscopy. A pump is used to perfuse a microdialysis probe with Ringer’s solution, which is fed to a flow cell to determine intratumor concentrations in real time to within a few *μ*M. The success and limitations of these techniques are identified, and suggestions are made as to further development. To the authors’ knowledge these are the first attempts made to quantify Mitomycin C concentrations in tumor tissue.

## Background

Bladder cancer is the seventh most common cancer in men. In 2008, approximately 386 300 cases were diagnosed and 150 200 patients died of the disease worldwide [1]. Approximately 70% of bladder tumors are superficial (not muscle invasive) at the moment of diagnosis. This includes Ta tumors, limited to the mucosa; carcinoma in situ, flat lesions limited to the mucosa with high risk of progression; and T1 tumors, invading the connective tissue (lamina propria) immediately under the mucosa. Patients with Ta tumors are treated with surgical resection (TURBT) followed by intravesical instillation with the chemotherapeutic Mitomycin C (MMC, approximately 2.4 mM) to kill remnant tumor cells in the bladder. Mitomycin C diffuses mainly into the mucosa but poorly deeper into lamina propria during intravesical therapy, and this may explain why MMC has poor efficacy treating tumors growing deeper in the bladder wall than Ta tumors.

Electroporation (EP) is a physical method in which short electric pulses are used to increase the permeability of the cell membrane, thereby allowing the introduction of molecules (e.g. drugs or genes) into the cytoplasm. It is the basis of electrochemotherapy, where uptake of chemotherapeutics is enhanced and their effect improved [2]. This modality is efficiently used in *vivo* for the treatment of cutaneous and subcutaneous tumors [3-6] and is now being introduced for the treatment of internal tumors [7-10]. A recent study investigated the cytotoxicity of the combination of EP and MMC on a bladder cancer cell line showing additive effect with an increase of 30% in cytotoxicity [11]. Data have shown that the combination of EP and MMC is more effective and abiding than MMC alone on the nude mice tumor model using a bladder cancer cell line [12]. It remains to be shown whether the intracellular concentration of MMC actually increases with EP, or whether the additive effect is due to the combined toxic effects of electric pulses and MMC.

Di Stasi *et al.* suggested that the application of electric current during the administration of MMC would improve the transport of the drug through the urothelium into the deeper layers of the bladder wall [13]. In that ex-vivo study, the bladder specimens were homogenized followed by HPLC analysis to obtain concentration-depth profiles. While functional, this method does not provide a clear picture of how the drug is penetrating the various tissue layers in the bladder wall.

The aim of this work is to develop methods for the detection and quantification of MMC directly in tissue, so it can be used in further studies. We used the nude mouse animal model aiming to measure MMC within tumors during treatment with MMC, quantify MMC uptake into tumor cells, and monitor the chemical contents of the tumor in real-time, as well as post-treatment inspection of mounted tumor samples. In the first effort, a novel method of UV-Vis absorption microscopy was employed in an attempt to directly map the concentration profile of the MMC within the tumor. This consists not only of the development of the imaging method itself, but also a number of adjustments to the sample preparation protocol. In the second effort, a form of on-line UV-Vis absorption spectroscopy fed by a microdialysis probe is used to directly monitor the internal contents of a mouse tumor during the MMC treatment. More accurate measurement of the pharmacokinetics may facilitate a mechanistic understanding of this treatment, potentially improving it to become a minimally invasive alternative to cystectomy (removal of the bladder) for patients with bladder tumors that invade the lamina propria (T1).

## Results and discussion

### UV-Vis Absorption microscopy

#### MMC Imaging Proof-of-concept

**Figure 1:**
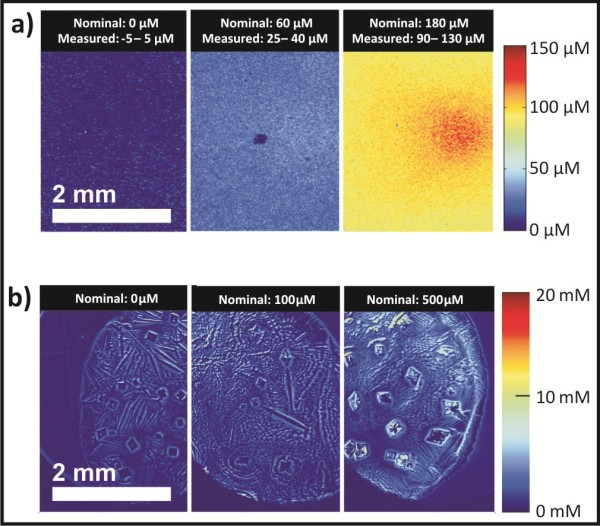
**Concentration micrographs of MMC in gelatin and in dried state. a)** Mitomycin C was dissolved in aqueous gelatin at concentrations of 0, 60, and 180 *μ*M to form thin hydrogel slices, that were imaged at the detection and reference wavelengths, respectively. The resulting images were used for computing the displayed concentration micrographs. **b)** Droplets of MMC were placed on a glass slide at the nominal concentrations and allowed to dry. Crystallite boundaries scatter light and appear as high MMC concentrations in the micrograph. The color scale shows the MMC concentrations at specific locations. The difference in units (*μ*M and mM) in the two color scales should be noted.

Initial imaging was performed on samples of gelatin spiked with MMC as a proof-of-concept. Samples of 100 *μ*m thick gelatin slabs (5% w/v in water) with 0 *μ*M, 60 *μ*M, and 180 *μ*M MMC were imaged, shown in Figure 1a. The gelatin samples were optically clear to the eye, but the difference in MMC concentration could be correctly calculated through image analysis. The calculated MMC concentrations depended linearly on the added concentrations as expected (data not shown), but required a calibration factor. Concentration variations across the micrographs in Figure 1a are ascribed to inhomogeneous sample illumination. Following the gelatin samples, drops of phosphate buffered saline containing 0 *μ*M, 100 *μ*M, and 500 *μ*M were placed on a microscope slide and dried in a vacuum desiccator. This caused salt crystals to form in place of the saline drops, with solid MMC mixed in with the salt. Figure 1b shows that sharp discontinuities in refractive index (at the air/solid interfaces) in the dried medium lead to scattering which manifests as an apparent absorption. Despite the artifacts at the crystal edges, higher concentration of MMC are clearly observed within the crystals in the image of 500 *μ*M concentration. These proof-of-concept images confirmed that the microscope setup, acquisition software, and image processing code could adequately image MMC in thin sample volumes.

#### Paraffin-embedded tumor samples

**Figure 2:**
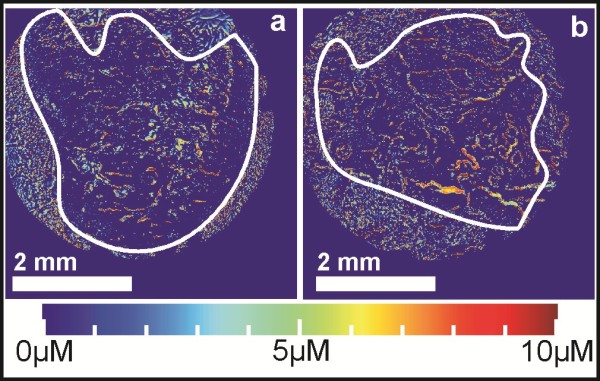
**Concentration micrographs of wax-embedded tumor samples.** Concentration micrographs of 10 *μ*m thick tumor samples embedded in paraffin wax after **a)** not being treated, and **b)** being treated with a 5 mM MMC injection before removal. The white line denotes the outer boundary of the tissue area, while the color map indicates the concentration of MMC at specific locations. Since only 5 mM MMC was injected, any concentrations higher than this are purely artifacts caused by light scattering.

Imaging attempts were next made on paraffin-embedded mouse tumors. Slices of thicknesses 100 *μ*m and 50 *μ*m were found to have too high absorbance for the analysis setup, in agreement with the tumor samples having a white appearance due to strong light scattering. Best results were obtained with 10 *μ*m thick tumor samples, while usable but non-optimal results were observed with 20 *μ*m thick samples. Figure 2 presents representative images of tumor sample slices without or with MMC treatment prior to removal. In contrast to images with thicker samples (20-100 *μ*m), the images acquired for 10 *μ*m thick samples are highly reproducible thanks to improvements made to the image processing code and microscope setup, as explained in the Methods section. Unfortunately there was no measurable difference between the treated and untreated tumors in terms of MMC concentration. Scattering can be seen from the tissue matrix, but no parts of the samples display increased levels of MMC.

#### Freeze-dried tumor samples

Following removal from the mouse, the tumor is immersed in a series of ethanol/water baths of increasing ethanol concentration to dehydrate the tissue, before being embedded in paraffin wax. Since MMC is highly soluble in both water and ethanol at the injected concentration (5 mM), we suspected that the MMC was being removed during this dehydration procedure. Thus, the sample preparation procedure was altered to eliminate solvent-induced dehydration. Subsequent samples were placed in liquid nitrogen immediately following removal from the mouse, and sectioned and stored at temperatures below -20°C until freeze-drying.

**Figure 3:**
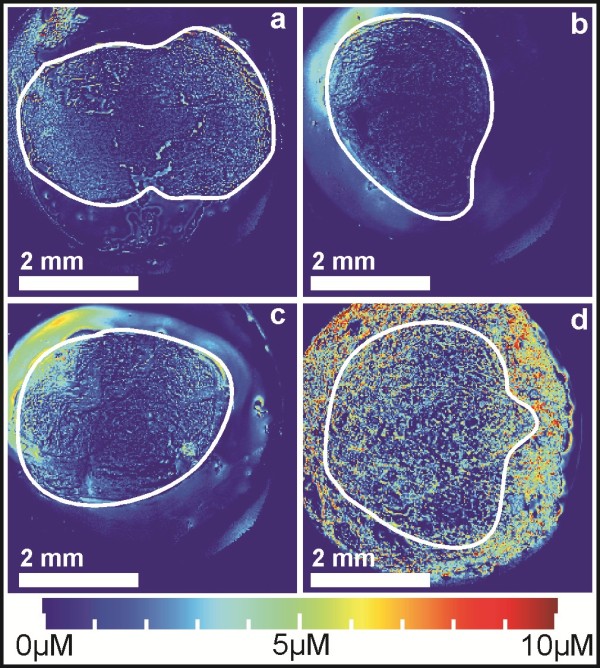
**Concentration micrographs of freeze-dried tumor samples.** Concentration micrographs of 10 *μ*m thick tumor samples frozen after removal and freeze-dried. The figure depicts samples that have **a)** not been treated, **b)** been treated with MMC, **c)** been treated with MMC and had electroporation performed, and **d)** have been spiked with MMC after freeze-drying. The white line denotes the outer boundary of the tissue area.

The freeze-drying process used frozen tumor slices attached by immersion oil to a glass slide, and was very effective at removing all water from the tissue samples. However, this process proved to be too mechanically stressful for the tissue samples, as several were damaged by the partial evaporation of the immersion oil under vacuum conditions (results not shown). Additionally, Figure 3 shows that there is no discernible difference between untreated (Figure 3a), treated (Figure 3b), and electroporation-treated (Figure 3c) samples. These same samples were spiked with 500 *μ*M MMC, dried, and re-imaged (Figure 3d). Even when large amounts of MMC were added to the sample, we observed very low concentrations in the calculated concentration micrographs.

An optimized procedure used vacuum-compatible silicone oil instead of vacuum-incompatible immersion oil for mounting the samples, and the samples were further positioned so that contact between the tumor slice and the mounting oil was avoided. The freeze-drying process was once again highly effective, but the tissue became a network of solid fibers. Since all areas of the sample were now either air gaps or fully opaque solid material, light absorption by MMC could not be detected on any sample area (data not shown).

### Microdialysis

#### Probe installation

The tumors were found to be extremely dense, making installation of the probe difficult. A 1.2 mm needle was used to produce a hole in the tumor for the guide cannula to fit, into which the probe was inserted.

#### Real-time monitoring of tumor contents

**Figure 4:**
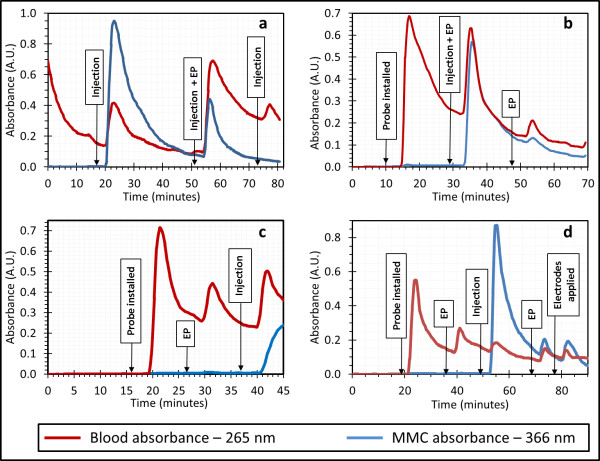
**Real-time monitoring of intratumor MMC via microdialysis.** All plots show the evolution of the magnitude of the MMC (blue line) and blood absorption (red line). There is a latency time of 4-5 min from the tissue fluid is sampled till it reaches the detector. In **a)** the probe is installed and the tumor is injected with MMC three times with and without electroporation (EP). In **b)** a second EP treatment is performed after the initial treatment, and a small MMC signal is observed, despite no additional MMC being injected. In **c)** an EP treatment without an MMC injection shows that EP alone does not produce an MMC signal. Plot **d)** shows an EP treatment without MMC injection, as well as the probes being placed on the tumor without application of voltage pulses. This shows that it is the physical manipulation of the tumor that is transferring MMC within the tumor, and that the measured MMC signal is unrelated to EP.

Following calibration and installation of the probe, the effect of injected MMC was observed. Figure 4a is a representative plot of the evolution of the MMC and blood signals during the experiment. Responses in the absorption signals were reproducibly and expectedly observed with a delay of 4-5 min after a given manipulation of the tumor due to the time required for the probe dialysate to reach the detection cell. The blue line represents the relative concentration of the MMC, while the red line represents that of the blood. Two observations are immediately obvious from this plot. First, the blood signal rises with the injection of MMC. While MMC has absorption components overlapping the blood absorption, this accounts for only 10% of the observed increase in the blood signal. We hypothesize that the manipulation of the tumor and the increased intratumoral pressure during injection forces fresh blood or extracellular fluid to the probe site where blood or fluid components are taken up by the probe, followed by an associated spike in this signal and a slow decline as equilibrium returns.

Second, no MMC concentration spike is observed after the third injection. Subsequent trials and tumor dissections led us to believe that the first injection creates the greatest pressure within the tumor, and thus opens paths in the tumor for injected volumes to escape. This results in subsequent injections passing out of the tumor without enough pressure being developed for liquid transport to the probe site. This was observed for every experiment when multiple injections were made (data not shown); thus the experiment was adapted to include only a single injection of MMC.

As shown in Figure 4b, a second spike in MMC concentration can be produced by applying an electroporation treatment sometime after the MMC injection. Figure 4c confirms that this is not some artifact of the EP treatment; such treatment before the MMC injection results only in a blood absorption spike. Thus, the MMC contributing to the increased signal seen in Figure 4b can only have come from the injected volume itself. The remaining question was: where was this MMC stored between the time of injection and when it reappeared? Figure 4d suggests that the MMC collects in extracellular space within the tumor, as well as the space between the tumor and skin, since the MMC concentration can be altered by merely placing the electrodes on the tumor, i.e, even without applying EP voltage pulses. Thus, we find that any physical handling of the tumor causes enough internal pressure to rearrange the chemical contents of the tumor.

## Conclusions

This work has for the first time demonstrated two novel methods of quantifying MMC in tumor tissue, both of which have shown promise. Real-time monitoring of tumor contents via a microdialysis probe continuously feeding a UV-Vis flow cell was able to measure MMC over the span of several hours with a sampling interval of less than a minute. In this particular application, the chemotherapeutic was injected directly into the tumor. The method was able to identify approximate intratumor concentrations, but lacked the sensitivity to determine any additional amount of MMC that may have entered the tumor cells under the application of EP. Nonetheless, this method proved to be a simple and compact method for continuous monitoring of specific tumor contents. Absolute concentration measurements in internal volumes will additionally require an *in vivo* calibration that was not explored in this work.

Absorption microscopy has shown promising results when imaging liquid or gelatin samples containing Mitomycin C at concentrations as low as 60 *μ*M. However, when attempting to apply the technique to tumor tissue samples, a number of problems were encountered. Tumor tissue can be very optically dense, calling for a sample thickness of 10 *μ*m thick or less, and even then every optical interface scatters light quite strongly. The dehydration process normally used to preserve tissue samples is likely to wash away any MMC, leaving none to be measured. Freeze-drying was employed to remove the possibility of MMC-loss during typical dehydration procedures, but proved to leave the samples in a state where MMC could not be imaged at all. Our conclusion from this work is that imaging MMC in preserved tissue is extremely difficult. Since the only really successful imaging of MMC occurred in aqueous solution, we suggest future efforts to be directed towards imaging planar cell cultures, and live, wet tissue samples.

## Methods

### UV-Vis Absorption Microscopy

Ultraviolet-visible spectroscopy (UV-Vis) measures the intensity of light that passes through a sample, *I*_1_, compared to the incident light intensity, *I*_0_. If the absorbance of light is solely due to molecular absorption, the measured absorbance, *A*, is related to the total compound concentration at every wavelength, *λ*, by the Beer-Lambert Law (Equation 1):

(1) $$A_{\lambda }=\text{log}\left[\frac{I_{0}}{I_{1}}\right]=\varepsilon_{\lambda }\cdot c\cdot L$$

**Figure 5:**
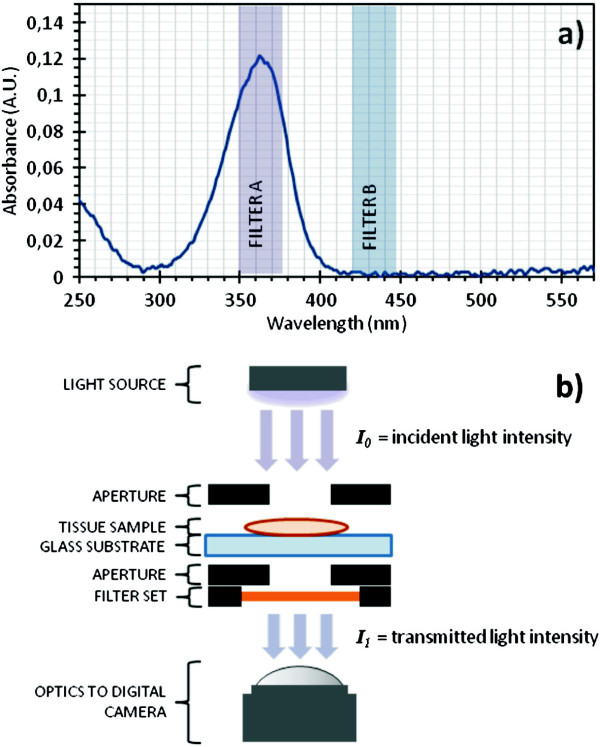
**MMC absorption spectrum and absorption microscopy setup. a)** Plot of the UV-Vis absorption spectrum of MMC, with highlighting of the transmission range of the filters used in imaging. **b)** Schematic of the UV-Vis microscopy setup, including placement of sample, filters, and apertures.

Thus, if one knows the molar extinction coefficient, *ε*_*λ*_, (determined empirically or found in literature), and the optical path length, *L*, one can easily determine the concentration, *c*, of the substance being examined. Figure 5a depicts the absorption spectrum of MMC.

Absorption microscopy works on the same principle as UV-Vis spectroscopy, but instead the concentration calculations are made for every pixel in an image. Certain bands of wavelengths are selected by using optical filters (as seen in Figure 5a), specifically chosen to match the peak absorption (filter A) and background absorption (filter B) when measuring MMC. Since absorption is additive, one can subtract the background absorption (filter B) from the peak absorption (filter A), to obtain the absorption contributions for MMC alone at each pixel in an image. Spatial mapping of concentration variations by optical absorption microscopy has been explored by Van Noorden *et al*. for quantifying enzymatic activity in rat liver tissue [14,15] with detailed descriptions of the methodology presented in a recent extensive review [16].

Referring to Equation 1 and the assumption of light absorbance being only caused by molecular absorption by the analyte of interest, this technique must balance two competing effects. First, a thicker sample (larger *L*) will allow more light to be absorbed at a given analyte concentration, thus allowing smaller concentrations to be detected. Second, thinner samples will place fewer structural artifacts (tumor cells, muscle, connective tissue) in the optical path, thus scattering less light. Light scattering on microscale features of tissue is governed by a combination of Mie and Rayleigh scattering, occurs at all wavelengths, and cannot be compensated or modeled. The light lost will appear as an apparent absorption and reduce the accuracy of our measurements. Thus, this technique must strike a balance between these effects to minimize scattering by artifacts (thinner samples) and maximize analyte absorption (thicker samples) to obtain accurate and reliable concentration micrographs. Additionally, the peak absorbance and background absorbance signals should be measured at the smallest useful separation in wavelength to minimize differential (wavelength dependent) light scattering between these two signals.

Absorption microscopy evaluation samples were prepared using gelatin (porcine skin, cat. no. G1890, Sigma-Aldrich, St. Louis, MO), phosphate buffered saline (Lonza, Basel, Switzerland), and MMC (Medac GmbH, Hamburg, Germany). Biopsy and tumor preparation was performed at Copenhagen University Hospital Herlev. Tumors were treated with MMC *in vivo* and then excised and dehydrated via subsequent immersions in solutions of 70% v/v, 96% v/v, and 99% v/v ethanol/water, respectively, and embedded in paraffin blocks. In the case of the freeze-dried samples, tumors were treated *in vivo*, excised, and immediately placed in liquid nitrogen, before being embedded in Tissue-Tek®; O.C.T™ Compound (Sakura, Alphen aan der Rijn, The Netherlands). Slices of paraffin or epoxy embedded samples were received on glass microscope slides, with the frozen samples needing to be placed on immersion oil or silicone oil since frozen slices will otherwise not stick to cooled glass slides. Two image sets were captured for each sample, one for the peak absorption (filter A, 365 nm bandpass) and one for the background absorption (filter B, 436 nm bandpass). Images were processed and evaluated by a custom MATLAB script with Equation 2 at its core:

(2) $$C=\frac{1}{\varepsilon_{\text{365nm}}\cdot L}\left(A_{\text{365nm}}-A_{\text{436nm}}\right)$$

where *C* is the matrix of concentration values across the image (i.e. pixels), the absorbance matrices *A*_*λ*_ are described by Equation 1, and *ε*_365nm_ and *L* are scalar values as in Equation 1 as well. The raw recorded images were additionally compensated for spatial variations in illumination intensity and effects of lens vignetting of the image projected onto the camera CCD chip, differences in CCD light sensitivities at the two wavelengths used, as well as dark noise of the CCD chip. The compensation procedure used images at the two wavelengths recorded without a sample in the light pathway, and with and without illumination light turned on. The resulting concentration micrographs are displayed as a color map to represent the presence of MMC.

Figure 5b shows a schematic of the microscope setup for imaging MMC in tissue samples. The apertures added on both sides of the sample plane function as light collimators that significantly reduce the collection of scattered stray light. An Axiovert 100M microscope (Carl Zeiss, Oberkochen, Germany) with an iXon digital camera (Andor Technology, Belfast, UK) was used for transmission imaging, illuminated by a 100 W halogen light source. Bandpass filters for 365 nm (filter A, band width 12 nm, model no. 365/12 BP) and 436 nm (filter B, band width 10 nm, model no. 436/10 BP) were used as received from DELTA (Hørsholm, Denmark). A custom machined stage and apertures were used, as was a custom made LabView program for controlling the microscope and the camera.

### Microdialysis

**Figure 6:**
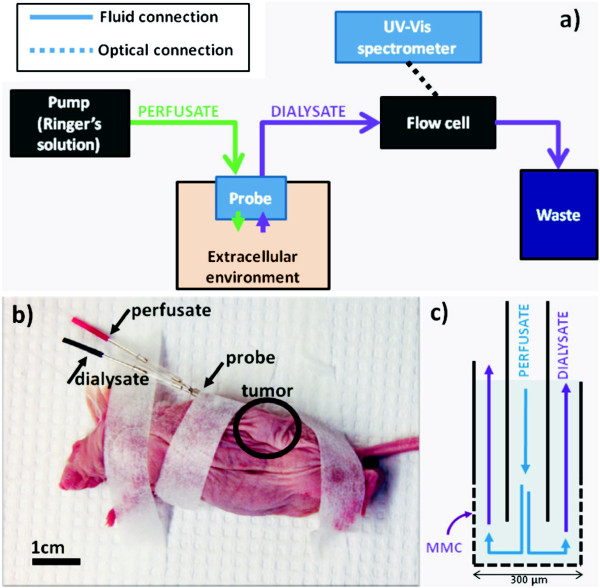
**Microdialysis setup and operation. a)** Ringer’s solution is perfused into the MD probe, and the sample is passed to a flow cell being continuously analyzed by a UV-Vis spectrometer. Absorption data is recorded and the dialysate continues to the waste container. **b)** The probe is inserted into the subcutaneous tumor on the mouse and fixed with surgical tape. **c)** The tip of the probe has Ringer’s solution continually flowing at a rate of 2 *μ*l/min, allowing small molecules (*e.g.* MMC) to pass into the dialysate and be carried to the flow cell for analysis.

A microdialysis probe works on the same principles as the Loop of Henle in the kidney. A carrier liquid, the perfusate, is perfused into the probe. This fluid travels to the probe tip and passes over the dialysis membrane. This membrane, which is in contact with the environment, allows chemical species (below a molecular weight of 10 kDa) to pass freely. The fluid, now called the dialysate, leaves the probe and travels to the measurement device. The construction of a microdialysis probe can be seen in Figure 6c. Since there is no convective transfer of fluid between the probe and the environment, this type of probe is particularly useful when measuring in small confined volumes or *in vivo*, for example monitoring accumulation of chemotherapeutics in breast tissue [17,18]. A typical dialysate sample is collected over 20-30 minutes (to accumulate a minimum volume) and analyzed using HPLC [19], capillary electrophoresis [17], or immunoassay methods [18]. For an overview of this technique please refer to the article by Chauruasia *et al.*[20].

The amount of chemical compound returned to the measurement system is a function of the pumping speed, probe environment, probe geometry, and properties of the compound itself. The concentration measured in the dialysate, under relatively steady-state conditions, can be simply expressed as a percentage of the chemical concentration in the probe’s immediate environment; this is called the extraction coefficient, and is determined experimentally via a calibration procedure before, during, or after the experiment. In the work presented here, an *in vitro* calibration was performed for every microdialysis probe used, not only to assess the extraction coefficient, but also to ensure that the probe was working within acceptable tolerances. The graphs showing *in vivo* concentrations over time are stated in absorbance units (AU) because no *in vivo* calibrations could be made during the time the probe was functional and installed in the mouse tumor. As as estimate of concentrations measured, *in vitro* calibration yielded 4±1×10^2^*μ*M/AU, tested for five different probes, and absorption resolution was better than 0.01 AU.

A schematic of the equipment configuration used for this experiment can be seen in Figure 6a. Microdialysis probes used in this experiment were type-AZ stainless steel, 4 mm membrane length (artificial cellulose) (cat. no. AZ-8-04, Eicom Corp., Kyoto, Japan), and are intended for use in rat brains. Perfusion of Ringer’s solution into the probe was accomplished with a single programmable syringe pump (New Era Pump Systems, Farminglade, NY, model no. NE-1000). The probe dialysate was fed into a fiber-optic micro-flow cell from World Precision Instruments (Sarasota, FL, model no. LWCC-M-10), with an optical path length of 10 mm accessed via quartz windows. An AvaSpec-3648 UV-Vis spectrometer using an AvaLight-DH-S-BAL combined deuterium-halogen light source (both from Avantes, Apeldoorn, The Netherlands) was connected to a laptop to acquire spectra and monitor specific wavelengths throughout the experiment. The spectrometer and light source were connected to the flow cell using 600 *μ*m core diameter, solarization-resistant optical fibers (1 m length, model no. QP600-1-SR-BX, Ocean Optics, Dunedin, FL).

*In vivo* experiments were conducted according to European Convention for the Protection of Vertebrate Animals used for Experimentation and with approval from the Danish Animal Experimentation Inspectorate. SW780 human bladder cancer cells were tested by rapid MAP27 panel (Taconic, Hudson, NY) without signs of infection. Cells were grown in DMEM culture medium (Gibco, Life Technologies, Carlsbad, CA), 10% fetal calf serum (Gibco, Life Technologies), penicillin, and streptomycin at 37°C and 5% CO_2_. After harvesting cells, a total of 1×10^6^ cells per ml diluted in PBS were injected subcutaneously on the left flank of 9 - 11 week-old NMRI-Fox1nu mice (own breeding). Tumor volume was calculated as *ab*^2^*π*/6 (*a*, largest diameter; *b*, largest diameter perpendicular to *a*). At an average tumor volume of 100 mm^3^, the mice were selected for the experiments. Hypnorm-dormicum (VetaPharma, Leeds, UK and Roche, Basel, Switzerland) was used for anesthesia complemented with rimadyl (Pfizer, New York City, NY). The mice were fixed with tape and intratumoral injections were performed with MMC at different concentrations, with volumes equivalent to the tumor volume. MMC was previously diluted to the desired concentration with isotonic saline. EP was performed using a 6 mm plate electrode and a square wave electroporator (Cliniporator, IGEA, Frankfurt, Germany). EP parameters were: 8 pulses of 100 *μ*s at 1.0 kV/cm and 1 Hz. Once the experiment was finished, the mouse was sacrificed. Figure 6b depicts the anesthetized mouse with a microdialysis probe installed.

## Abbreviations

AU: Absorbance units; CCD: Charge-Coupled Device; EP: Electroporation; HPLC: High performance liquid chromatography; MD: Microdialysis; MMC: Mitomycin C; PBS: Phosphate buffered saline; TURBT: Transurethral resection of bladder tumor; UV-Vis: Ultraviolet-visible spectroscopy.

## Competing interests

All authors declare that there are no competing interests.

## Authors’ contributions

LMF, JLV, GGH, and NBL participated in the conception and design of the study. LMF participated in design of the experimental setups and animal experiments, performed data acquisition and analysis, and participated in interpretation of results. JLV performed all animal experiments, and participated in data acquisition and interpretation. GGH and JG have participated in the interpretation of results. NBL conceived the idea of MMC quantification by UV-Vis absorption microscopy, and contributed to the experimental design, data analysis procedures, and results interpretation. All authors participated in the drafting of the manuscript. This manuscript has been read and approved by all authors.
